# Intact p53-Dependent Responses in miR-34–Deficient Mice

**DOI:** 10.1371/journal.pgen.1002797

**Published:** 2012-07-26

**Authors:** Carla P. Concepcion, Yoon-Chi Han, Ping Mu, Ciro Bonetti, Evelyn Yao, Aleco D'Andrea, Joana A. Vidigal, William P. Maughan, Paul Ogrodowski, Andrea Ventura

**Affiliations:** 1 Cancer Biology and Genetics Program, Memorial Sloan-Kettering Cancer Center, New York, New York, United States of America; 2 Weill Cornell Graduate School of Medical Sciences, Cornell University, New York, New York, United States of America; Cincinnati Children's Hospital Medical Center, United States of America

## Abstract

MicroRNAs belonging to the miR-34 family have been proposed as critical modulators of the p53 pathway and potential tumor suppressors in human cancers. To formally test these hypotheses, we have generated mice carrying targeted deletion of all three members of this microRNA family. We show that complete inactivation of miR-34 function is compatible with normal development in mice. Surprisingly, p53 function appears to be intact in miR-34–deficient cells and tissues. Although loss of miR-34 expression leads to a slight increase in cellular proliferation *in vitro*, it does not impair p53-induced cell cycle arrest or apoptosis. Furthermore, in contrast to p53-deficient mice, miR-34–deficient animals do not display increased susceptibility to spontaneous, irradiation-induced, or c-Myc–initiated tumorigenesis. We also show that expression of members of the miR-34 family is particularly high in the testes, lungs, and brains of mice and that it is largely p53-independent in these tissues. These findings indicate that miR-34 plays a redundant function in the p53 pathway and suggest additional p53-independent functions for this family of miRNAs.

## Author Summary

MicroRNAs (miRNAs) are small, non-coding RNAs that broadly regulate gene expression. MicroRNA deregulation is a common feature of human cancers, and numerous miRNAs have oncogenic or tumor suppressive properties. Members of the miR-34 family (miR-34a, miR-34b, and miR-34c) have been widely speculated to be important tumor suppressors and mediators of p53 function. Despite the growing body of evidence supporting this hypothesis, previous studies on miR-34 have been done *in vitro* or using non-physiologic expression levels of miR-34. Here, we probe the tumor suppressive functions of the miR-34 family *in vivo* by generating mice carrying targeted deletion of the entire miR-34 family. Our results show that the miR-34 family is not required for tumor suppression *in vivo*, and they suggest p53-independent functions for this family of miRNAs. Importantly, the mice generated from this study provide a tool for the scientific community to further investigate the physiologic functions of the miR-34 family.

## Introduction

The tumor-suppressor protein p53 is a master regulator of the stress response and provides a key barrier to cellular transformation and tumorigenesis [1]. Upon oncogene activation, DNA damage, and other forms of cellular stress, p53 accumulates in the nucleus where it induces or represses the transcription of a myriad of genes. Ultimately, p53 activation results in cell cycle arrest, apoptosis, or senescence, depending on the cellular context and the type of stimulus [2]. Although transcription-independent mechanisms have been reported [3], p53 mainly acts as a transcription factor for a large array of downstream effectors [4], including the proapoptotic proteins Puma, Noxa, and Bax, as well as the cell cycle inhibitor, p21 [5]–[11]. The essential tumor-suppressive function of p53 is further highlighted by the observation that this pathway is inactivated in the vast majority of human cancers [1], [12].

**Figure 1:**
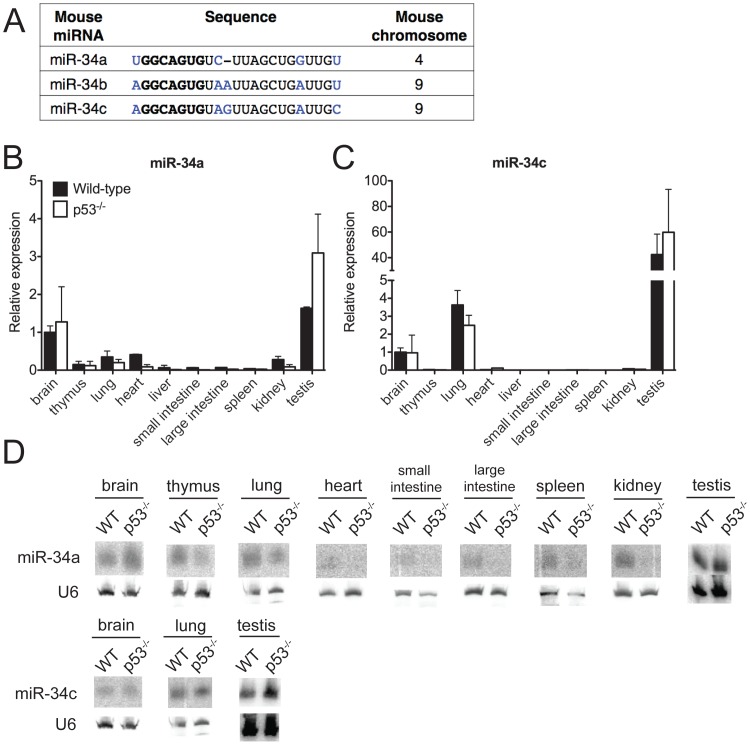
MiR-34 expression in wild-type and p53^−/−^ mouse tissues. (A) Sequence alignment of mouse miR-34a, miR-34b and miR-34c. Differing nucleotides are colored in blue. The seed sequences are in bold. (B–D) MiR-34a and miR-34c expression as detected by qPCR (B,C) and by Northern blotting (D) in tissues of wild-type and p53^−/−^ mice.

Several groups have recently suggested that miRNAs are also components of the p53 pathway. In particular, three highly related miRNAs—miR-34a, miR-34b, and miR-34c (Figure 1A)—are directly induced upon p53 activation in multiple cell types and have been proposed to modulate p53 function [13]–[20]. The precursors of these miRNAs are transcribed from two distinct loci: the miR-34a locus on chromosome 1p36 and the miR-34b∼c locus on chromosome 11q23. Canonical p53-binding sites are located in the promoter regions of both miR-34a and miR-34b∼c, and these miRNAs are *bona fide* direct transcriptional targets of p53 [13], [17], [18].

Ectopic expression of members of the miR-34 family is sufficient to induce cell cycle arrest or apoptosis, depending on the cellular context [14], [17]–[21]. Furthermore, loss-of-function studies using miR-34 antagonists have provided some evidence that this miRNA family is required for p53 function [13], [18], [22]–[24]. Many of the predicted miR-34 target genes encode for proteins that are involved in cell cycle regulation, apoptosis, and growth factor signaling. These include Cyclin E2, cMyc, MET, BCL-2, SIRT1, and members of the E2F family of transcription factors [13], [17], [17], [23], [25].

Consistent with a possible tumor-suppressor role, loss of expression of members of the miR-34 family has been reported in human cancers. Hemizygous deletion of the chromosomal region containing the miR-34a locus has been described in neuroblastomas and pancreatic cancer cell lines [14], [21]. Similarly, loss of 11q23, containing the miR-34b∼c locus, has been reported in prostate cancers [26]. Epigenetic silencing of miR-34 members has also been reported in human cancers. Promoter hyper-methylation of miR-34a is observed in non-small-cell lung cancers and melanomas [27], [28], and silencing of miR-34a and miR-34b∼c has been described in human epithelial ovarian cancers [29].

Although these observations point towards an important role for miR-34 members as critical downstream effectors of p53 and potential tumor suppressors, these hypotheses have not been formally tested using miR-34-deficient animals and cells. One notable exception is a recent elegant paper by Choi and colleagues demonstrating that miR-34-deficient MEFs are more susceptible to reprogramming [30]. However, the consequences of miR-34 loss on p53 function were not examined in detail.

Here we report the generation of mice carrying targeted deletion of all three members of the miR-34 family and systematically investigate the impact of miR-34 loss on the p53 pathway. We show that complete genetic inactivation of miR-34 does not detectably impair the p53 response in a variety of *in vivo* and *in vitro* assays. These findings highlight likely redundancies among p53's downstream effectors, show that the miR-34 family is largely dispensable for p53 function *in vivo*, and suggest possible p53-independent functions.

## Results

### p53-dependent and p53-independent miR-34 expression *in vivo*

To investigate the biological functions of miR-34, we first examined the expression of this family of miRNAs under basal conditions and in response to p53 activation *in vivo*. Under basal conditions, miR-34a and miR-34b∼c expression is particularly intense in the testis, brain, and lung of adult mice (Figure 1B–1D). MiR-34b∼c expression seems largely restricted to these three tissues, while miR-34a is detectable, albeit at lower levels, also in a variety of other organs (Figure 1B–1D).

**Figure 2:**
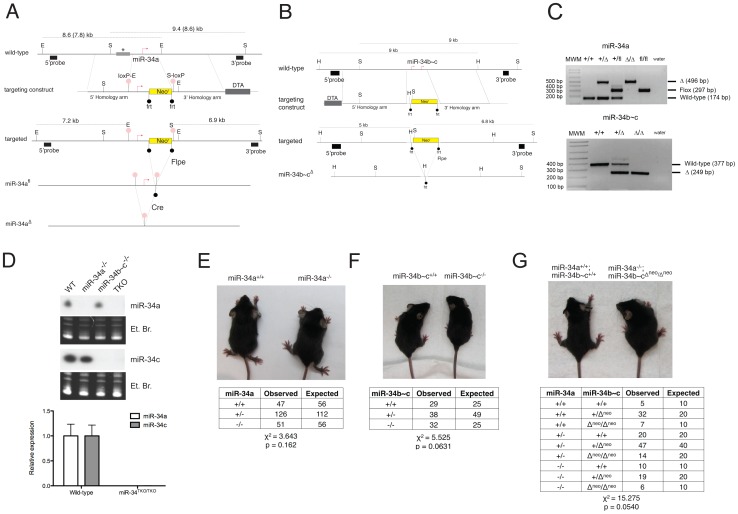
Targeted deletion of miR-34a and miR-34b∼c. (A) Targeting and screening strategy for the generation of constitutive and conditional miR-34a KO alleles. The restriction sites used for the Southern blot screening are indicated (S = SphI, E = EcoRI). The gray bar with an asterisk represents a genomic region absent in the 129SvJae strain but present in the C57BL/6 strain, which results in two distinct sizes in digestions. (B) Targeting and screening strategy for the generation of miR-34b∼c KO allele (H = HindIII, S = SpeI). (C) Genotyping by tail genomic PCR showing germline transmission of the miR-34a deleted and floxed alleles (upper panel), and the miR-34b∼c deleted allele (lower panel). (D) Northern blotting (upper panel) on total RNA extracted from the testes of mice with the indicated genotypes. Probes specific for miR-34a and miR-34c were used. Complete loss of miR-34a and miR-34c expression was further confirmed in MEFs by qPCR (lower panel). Representative pictures of miR-34a^−/−^ (E), miR-34b∼c^−/−^ (F), and miR-34^TKO/TKO^ (G) males at 4 weeks of age. The table below each picture summarizes the expected and observed frequencies of mice of each genotype as obtained from heterozygous inter-crosses. For the miR-34^TKO^ allele (G), double heterozygous mice were inter-crossed.

Consistent with previous reports indicating that miR-34a expression is under the direct control of p53 [13], [17], [18], we detected reduced levels of this miRNA in a subset of p53-deficient tissues (heart, small and large intestine, liver and kidney), but the levels of both miR-34a and miR-34b∼c remained high in the brains, testes and lungs (Figure 1B–1D) of p53^−/−^ mice, a finding that suggests that p53-independent mechanisms determine basal miR-34 transcription in these tissues. These results were obtained using two independent techniques: quantitative real time polymerase chain reaction (qPCR) and Northern blotting. The specificity and sensitivity of these assays were validated using miR-34-deficient mice as controls (Figure 1B–1D and Figure 2D).

Exposure to ionizing radiation, which leads to p53 stabilization and transcriptional activation, resulted in substantial miR-34a induction in the spleen, thymus, small and large intestine of wild-type mice, but not in the other tissues examined (Figure S1). We also observed modest but significant miR-34c induction in the thymus, small and large intestine of irradiated mice, but not in the other tissues examined.

### Generation of miR-34-deficient mice

To investigate the physiologic functions of the miR-34 family and to determine the extent to which its induction is required for p53 function, we generated mice carrying targeted deletion of both miR-34a and miR-34b∼c loci (Figure 2A–2C). To allow temporally and spatially restricted deletion, we also generated a conditional miR-34a KO allele (miR-34a^fl^, Figure 2A). Northern blot and qPCR analysis confirmed the loss of expression of the respective miRNAs in homozygous mutant animals (Figure 2D). Importantly, homozygous deletion of miR-34a did not lead to compensatory up-regulation of miR-34b∼c, and vice versa (Figure 2D and data not shown). MiR-34a^−/−^ and miR-34b∼c^−/−^ single KO mice were viable and fertile and were obtained at the expected Mendelian frequency (Figure 2E, 2F).

The sequence similarity between the three miR-34 family members (Figure 1A), which share the same “seed”, suggests that they may be functionally redundant. To examine the consequences of complete loss of miR-34 function, we crossed miR-34a^−/−^ and miR-34b∼c^−/−^ mice to generate compound mutant animals carrying homozygous deletion of all three family members (miR-34^TKO/TKO^). Complete loss of miR-34 expression in miR-34^TKO/TKO^ animals was confirmed by Northern blot and qPCR (Figure 2D). MiR-34^TKO/TKO^ mice of both sexes were obtained at approximately the expected Mendelian frequency (Figure 2G), did not display obvious macroscopic defects (Figure S2), and were fertile (data not shown). A full histological examination (Figure S3), complete blood cell count (Figure S4), and serum chemistry analysis (Figure S5) did not detect any statistically significant defects in adult miR-34^TKO/TKO^ mice of both sexes. An analysis of the major myeloid and lymphoid populations of the bone marrow, spleen and thymus also did not reveal any statistically significant difference between wild-type and miR-34^TKO/TKO^ mice (Figure S6).

### P53-dependent cell cycle arrest in miR-34^TKO/TKO^ MEFs

Next, we sought to determine whether loss of miR-34 expression affects the p53 response *in vitro*. We focused on the three best-characterized p53-dependent processes: replicative senescence, response to DNA damage, and response to oncogene activation [31]–[35].

**Figure 3:**
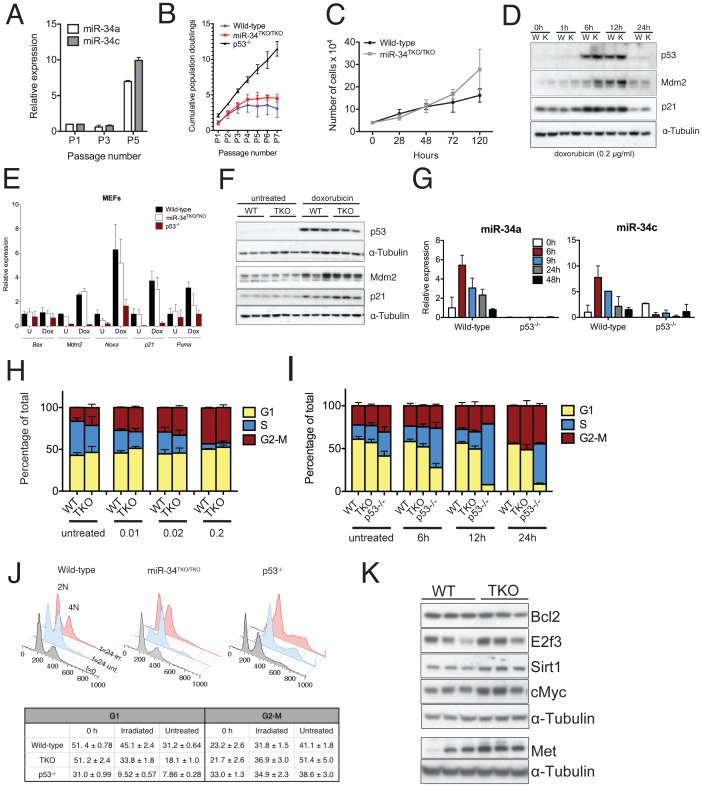
Response to p53 activation in miR-34^TKO/TKO^ mouse embryonic fibroblasts (MEFs). (A) MiR-34a and miR-34c expression in serially-passaged wild-type MEFs, as measured by qPCR. Error bars indicate 1 standard deviation (SD). (B) Cumulative population doublings of wild-type, miR-34^TKO/TKO^ and p53^−/−^ MEFs. Error bars indicate 1 SD. (C) Growth curves of wild-type and miR-34^TKO/TKO^ MEFs. Error bars indicate 1 SD. (D) Immunoblots of p53, p21 and Mdm2 in wild-type (W) and miR-34^TKO/TKO^ (K) MEFs treated with 0.2 µg/ml doxorubicin for the indicated time. (E) Expression of selected p53 targets in total RNA from doxorubicin-treated MEFs. Cells were treated with 0.2 µg/ml doxorubicin for 12 hours (Dox) or left untreated (U). Expression of the indicated genes was determined by qPCR. Error bars represent 1 SD. (F) Immunoblots showing p53 activation in three wild-type and three miR-34^TKO/TKO^ MEF lines. Cells were left untreated or treated with 0.2 µg/ml doxorubicin for 12 hours. (G) Time course of miR-34a and miR-34c expression in wild-type and p53^−/−^ cells treated with 0.2 µg/ml doxorubicin. MicroRNA expression was determined by qPCR. Error bars indicate 1 SD. (H, I) Cell cycle distribution of wild-type and miR-34^TKO/TKO^ MEFs. Asynchronously growing MEFs of the indicated genotype were treated with increasing doses of doxorubicin for 16 hours (H), or with 0.2 µg/ml doxorubicin for increasing time (I). Error bars indicate 1 SD. (J) Upper panel: cell cycle distribution of wild-type, miR-34^TKO/TKO^, and p53^−/−^ MEFs after 72 hours in starvation medium (gray histogram). Starved cells were released in complete medium containing colcemid and mock-treated (light blue histogram) or exposed to 20 Gy irradiation (red histogram). Cells were analyzed by 7-AAD staining at the indicated time after release in complete medium. Lower panel: percentages of irradiated and untreated cells in G1 and G2-M phases after 24 hours in complete medium. Experiments were performed on three independent wild-type and three independent miR-34^TKO/TKO^ MEF lines. (K) Immunoblot detection of predicted miR-34 targets on three independent wild-type and three independent miR-34^TKO/TKO^ MEF lines.

The ability to proliferate indefinitely is one of the hallmarks of cancer cells [36] and also one of the most striking consequences of p53 inactivation at the cellular level [35]. To investigate the role of miR-34 in replicative senescence, mouse primary fibroblasts (MEFs) derived from wild-type, p53^−/−^, and miR-34^TKO/TKO^ embryos were serially passaged. Although we detected a remarkable induction of miR-34a and miR-34c expression in late-passage wild-type MEFs compared to early-passage MEFs (Figure 3A), miR-34-deficient MEFs became senescent with a kinetic identical to wild-type MEFs (Figure 3B). This is in stark contrast with p53-deficient MEFs, which as expected proliferated indefinitely (Figure 3B). The only significant difference we observed was a slight but reproducible increase in the proliferation rate of early passage miR-34-deficient fibroblasts compared to controls (Figure 3B, 3C).

We next examined the role of miR-34 in the response to the DNA damaging agent doxorubicin. As previously reported [37], doxorubicin treatment leads to stabilization of p53 (Figure 3D) and up-regulation of its downstream targets p21 (Cdkn1a), Mdm2, Puma and Noxa (Figure 3D–3F). Expression of members of the miR-34 family was similarly upregulated in response to p53 stabilization (Figure 3G). Although as predicted, p53-null cells failed to arrest in G1 in response to doxorubicin treatment, the response of miR-34^TKO/TKO^ MEFs was indistinguishable from that of wild-type cells (Figure 3H–3I). Consistent with these results, doxorubicin treatment caused similar activation of p53 and of its downstream targets in wild-type and miR-34^TKO/TKO^ MEFs (Figure 3E and 3F).

The experiments described above were performed on asynchronously growing early-passage MEFs and as such may not be sensitive enough to detect a modest effect of miR-34 loss on the S-phase checkpoint. To measure cell cycle progression more accurately, we first synchronized MEFs by serum starvation and then released the cells in complete medium containing colcemid, a mitotic spindle inhibitor. With this experimental design, upon release in complete medium, cells synchronously proceed from G1 through S phase and then accumulate at the M (4N) phase. This assay provides a more sensitive way to determine the ability of cells to transition through the S-phase and allows detection of subtle defects in the DNA damage-induced S-phase checkpoint.

Although a reproducibly larger fraction of miR-34^TKO/TKO^ cells was able to transition through the S phase after ionizing radiation compared to wild-type MEFs (Figure 3J), we observed a similar difference in non-irradiated MEFs (Figure 3J). The most logical interpretation of these results is that miR-34-deficient MEFs, rather than being more resistant to irradiation-induced cell cycle arrest, possess a slightly faster basal proliferation or more rapid re-entry into the cell cycle following serum starvation. This interpretation is also consistent with the faster proliferation rate displayed by miR-34-deficient MEFs (Figure 3B, 3C) and with the observation by Lal and colleagues that miR-34a is involved in modulating the cellular response to growth factors [38].

We also examined the consequences of miR-34 loss in MEFs on the expression of a subset of its previously reported direct targets [17], [20], [23], [25]. We detected modest upregulation of cMyc, E2f3, Met and Sirt1 in miR-34-deficient MEFs, while Bcl2 was expressed at similar levels in wild-type and mutant cells (Figure 3K). The upregulation of Myc and E2f3 might contribute to the increased proliferation rate we have observed in miR-34 deficient MEFs.

### P53-dependent apoptosis in miR-34^TKO/TKO^ cells and mice

Having established that miR-34 is not required for cell cycle arrest in response to genotoxic stress in MEFs, we next sought to determine whether this miRNA family might contribute to p53-induced apoptosis.

**Figure 4:**
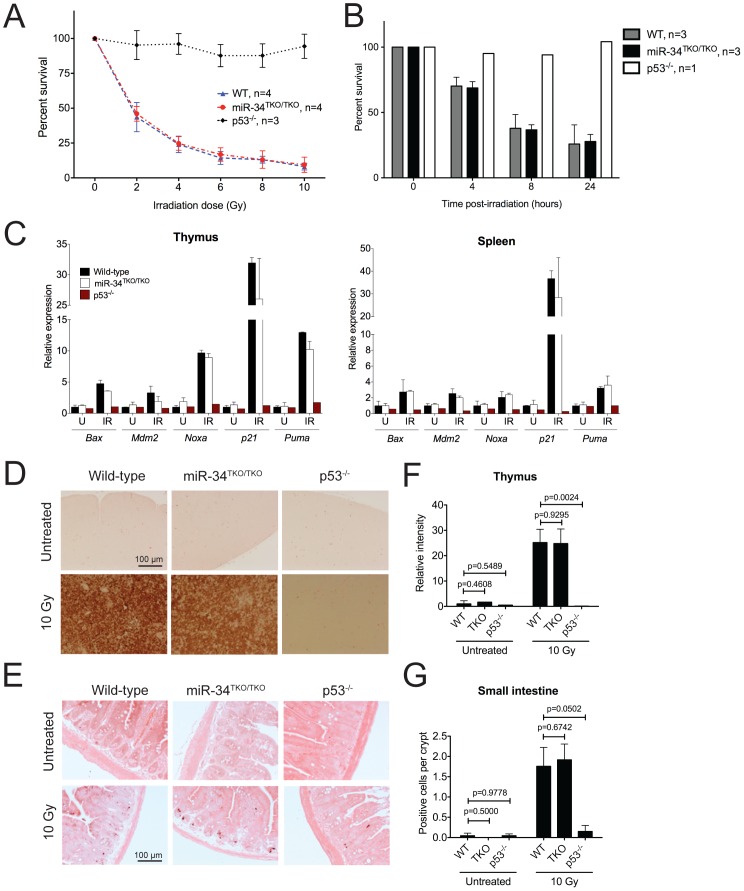
p53-dependent apoptosis in thymocytes and *in vivo*. (A) Percentages of viable wild-type, miR-34^TKO/TKO^, and p53^−/−^ thymocytes 16 hours after treatment with increasing doses of irradiation (0, 2, 4, 6, 8, and 10 Gy). Error bars represent 1 SD. (B) Percentages of viable wild-type, miR-34^TKO/TKO^, and p53^−/−^ thymocytes 4, 8, and 24 hours after irradiation (5 Gy). Error bars correspond to 1 SD. (**C**) Expression levels of p53 transcriptional targets in the thymi and spleens of untreated (U) and irradiated (IR, 10 Gy) wild-type, miR-34^TKO/TKO^ and p53^−/−^ mice by qPCR. (D, E) Representative cleaved caspase-3 immunohistochemistry of the thymus (D) and the small intestine (E) of untreated and irradiated (10 Gy) wild-type, miR-34^TKO/TKO^ and p53^−/−^ mice (n = 3 mice per group). Brown staining indicates cleaved caspase-3 (CC3). (F,G) Quantification of apoptosis in the thymus (F) and in the intestine (G) of control and irradiated animals. In panel F the relative staining intensity averaged over three microscopic fields per sample is plotted. In panel G, the average number of CC3-positive cells per crypt is plotted. At least 25 randomly selected crypts per sample were counted. Error bars correspond to 1 SD. P values were calculated using the two-tailed Student's t-test.

Thymocytes respond to ionizing radiations by rapidly undergoing apoptosis, an effect that is dependent on p53 [39]. We therefore examined the effects of DNA damage on thymocytes from wild-type, p53^−/−^, and miR-34^TKO/TKO^ mice. As expected, p53^−/−^ thymocytes were almost entirely resistant to irradiation-induced apoptosis; however, wild-type and miR-34-deficient cells were equally sensitive to DNA damage-induced apoptosis, as judged by dose-response and time-course experiments (Figure 4A, 4B).

To exclude the possibility that tissue culture conditions may have masked a physiologic role of miR-34 in modulating the p53 response, we next examined the consequences of p53 activation in miR-34-deficient tissues directly *in vivo*. Age- and sex-matched wild-type, miR-34^TKO/TKO^ and p53^−/−^ mice were exposed to 10 Gy of ionizing radiation and euthanized 6 hours later. Ionizing radiation induced similar activation of the p53 pathway and of its downstream effectors in wild-type and miR-34^TKO/TKO^ mice (Figure 4C). Analogous to what we observed in thymocytes *in vitro*, the apoptotic response was equally dramatic in wild-type and in miR-34-deficient mice, while it was virtually absent in p53^−/−^ animals (Figure 4D–4G).

Based on these results we conclude that miR-34 function is not required for p53-induced cell-cycle arrest and apoptosis in response to genotoxic stresses.

### miR-34 and tumor suppression *in vitro*

**Figure 5:**
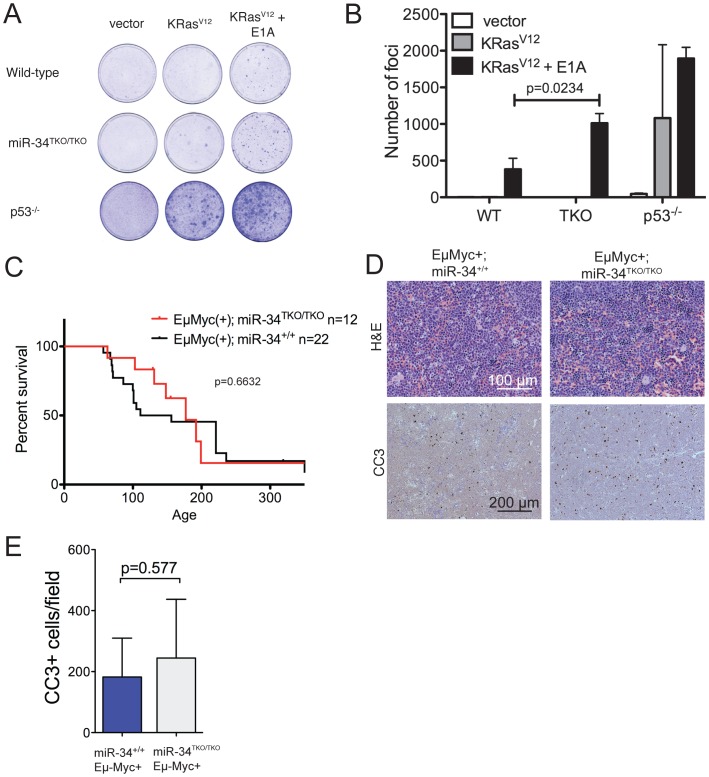
Oncogene-induced transformation in miR-34^TKO/TKO^ fibroblasts and mice. (A) Representative focus formation assays of wild-type, miR-34^TKO/TKO^, and p53^−/−^ MEFs. MEFS were infected with retroviruses expressing K-Ras^V12^ alone or K-Ras^V12^ and E1A. The results are representatitve of two independent experiments performed on a total of four wild-type and four miR-34^TKO/TKO^ MEF lines. (B) Bar plot showing the number of transformed foci. Error bars are 1 SD. (C) Survival curves of Eμ-Myc;miR-34^+/+^ and Eμ-Myc;miR-34^TKO/TKO^ mice. P-value was calculated using the log-rank (Mantel-Cox) test. (D) Histopathology and cleaved caspase-3 (CC3) immunohistochemistry of representative lymphomas obtained from Eμ-Myc;miR-34^+/+^ and Eμ-Myc;miR-34^TKO/TKO^ mice. (E) Bar plot showing the number of CC3-positive cells per low magnification field. Five Eμ-Myc;miR-34^+/+^ tumors and and four Eμ-Myc;miR-34^TKO/TKO^ tumors were analyzed. Error bars indicate 1 SD.

The p53 pathway provides a crucial barrier against the neoplastic transformation of primary cells [40]. Supra-physiologic proliferative stimuli, such as those caused by sustained oncogene activation, lead to transcriptional activation of p19Arf, which in turn results in stabilization and activation of p53, and consequently apoptosis or cell cycle arrest [41]. For example, ectopic expression of a constitutively active K-Ras (K-Ras^V12^) in wild-type MEFs leads to oncogene-induced senescence, but the concomitant inactivation of p53 is sufficient to allow full cellular transformation [31]. To test whether miR-34 plays a role in this context, we ectopically expressed oncogenic K-Ras in wild-type, miR-34^TKO/TKO^, and p53^−/−^ MEFs. As shown in Figure 5A, complete loss of miR-34 function was not sufficient to allow primary MEFs to be transformed by K-Ras^V12^ alone, while p53-deficient MEFs were readily transformed in the same assay. However, when MEFs were co-transduced with oncogenic K-Ras and E1A, which binds to and inhibits the retinoblastoma protein (pRb) [42], we observed a slight increase in the number of foci formed in miR-34^TKO/TKO^ MEFs compared to wild-type cells (Figure 5A, 5B). These results show that while miR-34 alone is not required for p53-mediated tumor suppression in MEFs, its loss might cooperate with inactivation of the Rb pathway in promoting cellular transformation.

### miR-34 and tumor suppression *in vivo*

To extend our analysis to an *in vivo* setting, we next examined whether miR-34 inactivation is sufficient to accelerate spontaneous and oncogene-induced transformation in mice. P53-deficient mice exhibit a high incidence of spontaneous tumors, in particular lymphomas and sarcomas [43]–[45], and p53 inactivation greatly accelerates tumor formation in a variety of mouse models of human cancer [46]–[51]. To determine whether loss of miR-34 expression leads to increased spontaneous tumorigenesis, we aged a cohort of 14 miR-34^TKO/TKO^ and 12 wild-type mice. The animals were monitored for at least 12 months (wild-type = 359 days; miR-34^TKO/TKO^ = 359 days) and up to 17.3 months (wild-type = 521 days; miR-34^TKO/TKO^ = 521 days). All wild-type and miR-34^TKO/TKO^ mice appeared healthy and miR-34^TKO/TKO^ mice did not show a reduction in life span compared to wild-type controls (Figure S7). For comparison, the median survival of p53^−/−^ mice has been reported to be 4.5 months and by 10 months of age all p53^−/−^ mice have died or developed tumors [45]. In addition, ∼40% of p53^+/−^ mice develop tumors by 16 months of age [45]. Thus, although a longer follow-up of miR-34^TKO/TKO^ mice may be needed to uncover very subtle defects in tumor suppression, we conclude that loss of miR-34 expression does not lead to a substantial increase in spontaneous tumorigenesis.

We next sought to determine whether loss of miR-34 might accelerate tumor formation in response to genotoxic stress. P53^−/−^ mice irradiated shortly after birth display accelerated tumorigenesis compared to non-irradiated littermates [52]. We therefore exposed a cohort of 14 miR-34^TKO/TKO^ and 11 wild-type mice to 1 Gy of ionizing radiation soon after birth and monitored them for 42–60 weeks. Both wild-type and miR-34-deficient mice appeared healthy throughout the follow-up period (Figure S7), in striking contrast with the ∼15 weeks reported median tumor-free survival of irradiated p53^−/−^ mice [52]. Although it will be important to follow a larger cohort of animals over a more prolonged period, these results suggest that miR-34 does not provide a potent barrier to tumorigenesis in response to genotoxic stress *in vivo*.

Finally, we sought to determine whether genetic ablation of miR-34 could contribute to tumor formation in cooperation with a defined oncogenic lesion. For these experiments, we chose the Eμ-Myc model of B cell lymphomas [53]. A crucial tumor-suppressive role for p53 is well established in this mouse model and inactivation of the p53 pathway results in greatly accelerated lymphomagenesis [46], [47], [54]. However, even in this context complete loss of miR-34 expression was not sufficient to accelerate tumor formation. The incidence and latency of B cell lymphomas was virtually identical in Eμ-Myc;miR-34^TKO/TKO^ and Eμ-Myc;miR-34^+/+^ mice (Figure 5C) and the resulting tumors displayed similar histopathological features and extent of spontaneous apoptosis (Figure 5D–5E).

## Discussion

We have reported the generation of mice carrying targeted deletion of miR-34a, miR-34b and miR-34c, and we have investigated the consequences of loss of miR-34 expression on p53-dependent responses *in vitro* and *in vivo*. Our results show that complete loss of miR-34 expression is compatible with normal development and that the p53 pathway is apparently intact in miR-34-deficient mice.

Our observation that inactivation of miR-34 does not impair p53-mediated responses *in vitro* and *in vivo* is particularly relevant because a key role for miR-34 in the p53 pathway had been previously proposed by a number of independent groups. The results presented in this paper do not necessarily conflict with previous experiments showing that ectopic expression of miR-34 can induce many of the most characteristic consequences of p53 activation; here we have tested whether miR-34 is necessary for p53 function and not whether it is sufficient.

More difficult, however, is to reconcile our findings with previous reports of impaired p53-function in cells treated with miR-34 antagonists. Because previous work has relied on the use of miRNA antagonists to inhibit miR-34 function, it is possible that some of the previous observations reflected miR-34-independent off-target effects.

It is also possible that other miRNAs sharing sequence similarities with miR-34 may compensate for miR-34 loss in the knock-out animals. In particular, members of the miR-449 family (miR-449a, b and c) have the same “seed” sequence as miR-34, and miR-34 antagonists could in principle impair their function as well. A conclusive test for this hypothesis will require the generation of compound miR-34 and miR-449 mutant animals, but several lines of evidence suggest that this explanation is not particularly likely. First, in the tissues and cells used in our experiments, the expression of miR-449 members is much lower compared to miR-34a and miR-34c, as judged by multiple independent methods including qPCR, Northern blotting and high throughput sequencing (Figure S8 and data not shown). A notable exception is represented by the testis, in which expression of miR-449a is particularly elevated (Figure S8). In addition, miR-449 expression is not substantially increased in miR-34-null mice, and activation of the p53 pathway does not lead to significant upregulation of miR-449 (Figure S8).

We would like to emphasize that our results do not necessarily indicate that members of the mIR-34 family are not components of the p53 pathway. Given the essential tumor-suppressive function exerted by p53, it is perhaps not surprising that multiple and partially redundant effector arms are recruited in response to its activation. It is plausible that the simultaneous inactivation of multiple effector arms is required to measurably impair p53 function. Consistent with this model is our observation that while loss of miR-34 expression alone does not allow the transformation of primary cells by oncogenic K-Ras, it slightly increases the efficiency of transformation when combined with inactivation of the Rb pathway by E1A (Figure 5A, 5B). In this context, it will be important to systematically probe the extent of functional cooperation between this family of miRNAs and other, previously characterized p53 effectors.

We also wish to point out that in this manuscript we have investigated the best-characterized functions of p53 (cell cycle arrest, apoptosis and tumor suppression) and it remains possible that miR-34 participates in other p53-dependent processes. For example, p53 has been proposed to modulate autophagy [55] and stem cell quiescence [56], [57] and we cannot exclude that miR-34 plays an important role in these contexts. Future studies using the miR-34-deficient animals we have generated will be needed to test these possibilities.

With respect to the potential tumor suppressive role of miR-34, our experiments indicate that loss of miR-34 expression does not lead to an obvious increase in tumor incidence in mice and does not cooperate with Myc in the context of B cell lymphomagenesis. However, the tumor suppressive function of miR-34 might be restricted to specific tissues and loss of miR-34 might cooperate with specific oncogenic lesions. In humans, for example, loss of miR-34 expression has been reported in a large fraction of primary melanomas, prostatic adenocarcinomas and small cell lung cancers [27], [28], among others. Introducing the miR-34-null alleles we have generated into mouse models of these types of human cancers will be important to fully explore the tumor suppressive potential of this family of miRNAs.

An additional issue raised by the results presented in this manuscript relates to possible p53-independent functions of miR-34. We show that under basal conditions the expression of both miR-34 loci is particularly elevated in the testes and, to a lesser extent, in the brains and lungs of mice. Importantly, in these three tissues, miR-34 expression is almost entirely p53-independent (Figure 1B–1D and [58]), a finding that suggests that additional transcription factors control the expression of this family of miRNAs in the absence of genotoxic or oncogenic stresses.

A role for miR-34c in spermatogenesis and in controlling the first zygotic cleavage has been recently proposed [58], [59]. Although our observation that single KO and miR-34^TKO/TKO^ mice produce viable offspring argues against an essential role for miR-34 in these processes, members of the related miR-449 family, that are particularly highly expressed in the testis (Figure S8), could partially compensate for miR-34 loss in this context.

Recent reports have also implicated miR-34 in neuronal development and behavior [60], [61] and a role for miR-34c in learning and memory [62], as well as in stress-induced anxiety [63], has been reported. In addition, inactivation of miR-34 expression has been recently shown to lead to accelerated neurodegeneration and ageing in *Drosophila melanogaster* [64]. A detailed behavioral and neuroanatomical analysis, as well as a careful characterization of the long-term consequences of miR-34-loss will be essential to confirm and extend these hypotheses in mice.

In conclusion, we have reported the generation and characterization of miR-34-deficient mice with a particular focus on the consequences of miR-34 loss on the p53 pathway. The genetically engineered mouse models described in this study will be essential to further investigate the physiologic functions and the tumor suppressive potential of this important miRNA family.

## Materials and Methods

### Generation of miR-34 constitutive and conditional knockout mice

The “recombineering” method [65] was used to modify a BAC clone (RP-23-410P10) containing the miR-34a locus to generate the miR-34a conditional knockout allele. A frt-Neo-frt-loxP cassette was first inserted ∼480 bp downstream of the pre-miR-34a sequence. Gap-repair was used to retrieve a 9.6 kbp fragment containing the frt-Neo-frt-loxP cassette, ∼4 kb of 3′ homology arm, and ∼3.7 kb 5′ homology arm, and including the pre-miR-34a sequence. The fragment was cloned into the targeting plasmid pKS-DTA, and a second loxP site was introduced into a unique KpnI site located ∼500 bp upstream of the pre-miR-34a sequence. The final targeting construct was linearized with NotI and electroporated into V6.5 murine embryonic stem cells (ESC). Following selection with G418, ESC colonies were isolated and screened by Southern blotting using DNA probes mapping outside the targeted region. Two targeted clones were expanded and injected into C57BL/6 blastocysts to generate chimeric mice. High contribution chimeras were subsequently crossed to Actin-flpe transgenic mice [66] to excise the frt-Neo-frt cassette and generate the miR-34a conditional knockout allele (miR-34a^fl^) or crossed to CAG-Cre mice [67] to excise the entire region flanked by the loxP sites and obtain the constitutive miR-34a KO allele (miR-34a^Δ^). Lastly, miR-34a^+/fl^ and miR-34a^+/−^ were intercrossed to obtain miR-34a^fl/fl^ and miR-34a^−/−^ animals.

To generate mice carrying deletion of the miR-34b∼c bicistronic cluster, we used recombineering to replace a 1.3 kbp DNA region in BAC RP-23-281F13 containing pre-miR-34b and pre-miR-34c with a frt-Neo-frt cassette. A 8.4 kbp DNA fragment containing the frt-Neo-frt cassette, the 3.7 kbp 5′ homology arm, and 2.8 kbp of 3′ homology arm was retrieved from the engineered BAC and cloned into pKS-DTA. The resulting targeting vector was linearized by NotI and electroporated into V6.5 ESCs. Upon selection, two independent clones were injected into C57BL/6 blastocysts. High contribution chimeras were crossed to Actin-flpe transgenic mice for germline transmission of the targeted allele and to delete the Neo cassette resulting in the miR-34b∼c^Δ^ allele. The miR-34b∼c^+/−^ mice were intercrossed to obtain miR-34b∼c^−/−^ animals. The Eμ-Myc mice were generated and described by Adams and colleagues [53] and the p53^−/−^ mice were generated by Jacks and colleagues [44]. Genotyping protocols are provided in Text S1. All animal studies and procedures were approved by the MSKCC Institutional Animal Care and Use Committee. Mice were maintained in a mixed 129SvJae and C57BL/6 background. The miR-34a<floxed> mice and the miR-34b∼c−/− mice are available to the research community through The Jackson Laboratory (JAX Stock Numbers 018545 and 018546).

### Generation of MEFs

Primary MEF lines were generated from E13.5 embryos using standard protocols. miR-34^TKO/TKO^ embryos were obtained by intercrossing miR-34 mutant mice. Wild-type MEFs were generated in parallel. p53^−/−^ embryos were obtained by intercrossing p53^+/−^ mice. Genotyping protocols are provided in Text S1. MiR-34 wild-type and miR-34^TKO/TKO^ MEF lines were also verified by qPCR.

### Northern blotting and qPCR

RNA extraction was performed by homogenizing tissues and cells in TRIzol reagent (Invitrogen) according to manufacturer's instructions. For Northern blotting, 15 µg of each RNA sample was loaded into a 15% Urea-PAGE gel and blotted onto a Hybond-N^+^ nylon membrane (GE Healthcare). The blots were then hybridized with ^32^P-labeled probes specific for miR-34a, miR-34c, and U6. qPCR was performed using primers and probes by Applied Biosystems according to manufacturer's instructions. Sno-135 was used for normalization.

### Cell culture and cell cycle analysis

Passage 2 or 3 primary MEFs were used for all experiments and cultured at 37°C (5% CO_2_) in DME-HG with 10% FBS (complete medium) or 0.1% FBS (starvation medium) supplemented with L-glutamine, penicillin, streptomycin, and β-mercaptoethanol. For BrdU cell cycle analysis, wild-type, miR-34^TKO/TKO^, and p53^−/−^ MEFs were plated in complete medium at 70% confluence, treated with varying doses of doxorubicin for 16 hours or treated at different time points, and pulsed with 10 µM BrdU for one hour. The BD Pharmingen APC-BrdU kit was used to process harvested samples and used according to manufacturer's protocol. For the irradiation experiments, 150,000 wild-type, miR-34^TKO/TKO^ and p53^−/−^ MEFs were seeded into each well of a 6-well culture plate and starved for 72 hours. MEF lines were then trypsinized and resuspended in complete medium and either irradiated (20 Gy, Cs-137 irradiator, Shepherd Mark-I) or left untreated. Cells were replated into complete medium containing 500 ng/ml colcemid at 70% confluence and harvested 24 h later. Samples were processed as mentioned above and stained with 7-AAD. Flow cytometry was performed using FACSCalibur (BD Biosciences), and data were analyzed using FlowJo software (TreeStar).

### Growth curves and 3T3 assay

Wild-type and miR-34^TKO/TKO^ MEFs were seeded into a 6-well plate (40,000 cells/well) and counted every day for the growth curves. The standard 3T3 protocol was followed to determine the cumulative population doublings of wild-type, miR-34^TKO/TKO^, and p53^−/−^ MEFs. Briefly, 3×10^5^ cells were seeded in a 6 cm^2^ dish and counted and passaged every three days.

### Thymocyte apoptosis assay

Thymocytes were isolated from sex-matched, age-matched wild-type, miR-34^TKO/TKO^, and p53^−/−^ mice and seeded at a density of 1×10^6^ cells/ml in MEF medium. Thymocytes were then treated with various doses of irradiation (2, 4, 6, 8, and 10 Gy, Cs-137 irradiator, Shepherd Mark-I) or left untreated. For the time course experiments, thymocytes were treated with 5 Gy of irradiation and harvested 4, 8 and 24 h after treatment. Samples were stained with AnnexinV and propidium iodide (Roche) according to manufacturer's protocol. Flow cytometry was performed using FACSCalibur (BD Biosciences) and data were analyzed using FlowJo software (TreeStar).

### Focus formation assay

Phoenix cells (Orbigen) were transfected using FUGENE 6 (Promega) with retroviral constructs of K-Ras^V12^ alone or together with E1A according to manufacturer's instructions. Wild-type, miR-34^TKO/TKO^, p53^−/−^ MEFs were seeded at 70% confluence and infected with virus. Plates were fixed with methanol and stained with crystal violet two weeks after infection. Foci were quantified using ImageJ.

### Western blotting and antibodies

Cells were lysed in RIPA buffer containing protease inhibitors. Proteins (25 µg) were separated on a NuPAGE Bis-Tris gel (Invitrogen), and transferred onto a PVDF membrane (Millipore). Blocking was performed with 5% milk in TBST. Primary antibodies used were anti-p21 (1: 1000, Santa Cruz, F-5), anti-Mdm2 (1∶1000, Abcam, 2A10), anti-Met (1∶1000, Millipore, 07-283), anti-Bcl2 (1∶500, Cell Signaling, #2876S), anti-E2f3 (1∶500, Millipore, PG37), anti-Sirt1 (1∶1000, Cell Signaling #2028), anti-cMyc (1∶1000, Cell Signaling, D84C12), and anti-α-Tubulin (Sigma, DM1A). The anti-p53 antibody (1∶300) was a kind gift of Kristian Helin (BRIC, Denmark). Secondary antibodies were obtained from Cell Signaling. ECL reagents were obtained from GE Healthcare. Western blot bands were quantified using ImageJ.

### Immunohistochemistry

Mice were irradiated with 10 Gy and sacrificed 6 hours after. PFA-fixed, paraffin-embedded sections were deparaffinized in xylene, and rehydrated. The samples were stained with Cleaved Caspase-3 antibody(Cell Signaling, #9664) overnight, according to Cell Signaling protocol. The samples were also counterstained with 0.1% alcoholic Eosin Y solution (Sigma-Aldrich) or 30% hematoxylin. The sections were then dehydrated and mounted in Permount (Fisher Scientific). Sample pictures were quantified using ImageJ.

## Supporting Information

Figure S1 Relative miR-34 expression in mouse tissues upon irradiation. (A) MiR-34a and miR-34c expression by Northern blotting under basal conditions and 18 hours after irradiation (10 Gy). (B) MiR-34a (left panel) and miR-34c (right panel) expression by qPCR under basal conditions and 24 h after irradiation (10 Gy). Expression levels of treated samples were normalized to untreated samples. Error bars are standard deviations.
(PDF)

Figure S2 Macroscopic characterization of miR-34^TKO/TKO^ mice. (A) Representative pictures of internal organs obtained from age- and sex-matched wild-type and miR-34-null adult mice. (B) Scatter dot plots showing total body weight of age-matched and sex-matched wild type (WT) and miR-34-null (TKO) mice at different ages (upper-left panel) and relative weight of internal organs (remaining panels; n≥6 per genotype). Error bars indicate mean +/−1 S.E.M. P-values were obtained using the unpaired two-tailed t-test.
(PDF)

Figure S3 Microscopic characterization of miR-34^TKO/TKO^ mice. Representative images of hematoxylin and eosin staining of heart, kidney, liver, lung, small intestine, ovary, testis, and spleen (black scale bar, 200 µm), brain (green scale bar, 2000 µm), and colon (red scale bar, 100 µm) from wild-type and miR-34^TKO/TKO^ mice.
(PDF)

Figure S4 Complete blood cell count of age- and sex-matched wild-type and miR-34-deficient mice. Peripheral blood samples obtained from sex- and age-matched adult (age range 3–16 months) wild-type (WT) and miR-34-null (TKO) mice were subjected to complete blood cell count (n≥5 per genotype). Error bars indicate mean +/−1 S.E.M. The P-value for each parameter was calculated using the two-tailed unpaired t-test. Abbreviations used: WBC = White blood cells; RBC = red blood cells; HCT = Hematocrit; HGB = Hemoglobin; MCV = Mean Corpuscolar Volume; MCH = Mean Corpuscolar Hemoglobin; MCHC = Mean Corpuscolar Hemoglobin Concentration.
(PDF)

Figure S5 Serum chemistry of age- and sex-matched wild-type and miR-34-deficient mice. Samples obtained from sex- and age-matched adult (age range 3–16 months) wild-type and miR-34^TKO/TKO^ mice were subjected to a standard panel of serum chemistry tests to determine liver and kidney function (n≥5 per genotype). Error bars indicate mean +/−1 S.E.M. The P-value for each assay was calculated using the two-tailed unpaired t-test. Abbreviations used: ALP = Alkaline Phosphatase; ALT(SGPT), Alanine Transaminase; AST = Aspartate transaminase; A/G Ratio = (Albumin/Globulin Ratio).
(PDF)

Figure S6 Bone marrow, spleen and thymus analysis of age- and sex-matched wild-type and miR-34^TKO/TKO^ mice. (A) Representative contour plots showing lymphoid and myeloid cell populations in the bone marrow, spleen, and thymus of age-matched and sex-matched wild-type (n = 6) and miR-34^TKO/TKO^ mice (n = 6). (B) Scatter dot plots summarizing the results of the analyses shown in (A). Error bars are +/−1 standard deviation. P-values were calculated using the two-tailed t-test.
(PDF)

Figure S7 Overall survival of wild-type and miR-34^TKO/TKO^ cohorts. (A) Survival curves for wild-type and miR-34^TKO/TKO^ mice. Age range of the cohorts is 359–521 days (mean: 464 days) for wild-type and 359–521 days (mean: 445 days) for miR-34^TKO/TKO^. P-value = 1 (log-rank test). (B) Survival curves for mouse cohorts with indicated genotypes irradiated with 1 Gy 2 days after birth. Age range of the cohorts is 298–425 days (mean: 333 days) for wild-type and 387–425 days (mean: 401 days) for miR-34^TKO/TKO^. P-value = 1 (log-rank test).
(PDF)

Figure S8 Expression of miR-449a, miR-449b and miR-449c. (A) Northern blot detection of miR-449a, miR-449b, miR-449c and miR-34a in a panel of tissues from wild type and miR-34^TKO/TKO^ mice. For each tissue, the same membrane was serially probed first for the three members of the miR-449 family and lastly for miR-34a. RNAs from miR-34^TKO/TKO^ tissues were included to control for cross-hybridization. Notice the loss of signal for miR-449b in the miR-34^TKO/TKO^ lung and testis samples, which likely reflects cross-hybridization of the miR-449b probe to miR-34. (B–D) qPCR detection of miR-449 family members in MEFs (B), thymus (C), and spleen (D) of wild-type, p53^−/−^ and miR-34^TKO/TKO^ mice exposed to DNA damaging agents. MEFs were treated with 0.2 µg/ml doxorubicin for 12 hours. Mice were irradiated with 10Gy and euthanized 6 hours later. Notice that the all three members of the miR-449 family are detected at significantly lower levels compared to miR-34, consistent with the Northern blot analysis shown in panel A.
(PDF)

Text S1 Supporting information texts. Genotyping protocols and supplementary methods are provided.
(PDF)
