# Supplementary figures and images for: Intact p53-Dependent Responses in miR-34–Deficient Mice
Source: PLoS Genet. 2012 Jul 26;8(7):e1002797. doi: 10.1371/journal.pgen.1002797 (PMC3406012; doi:10.1371/journal.pgen.1002797)

A

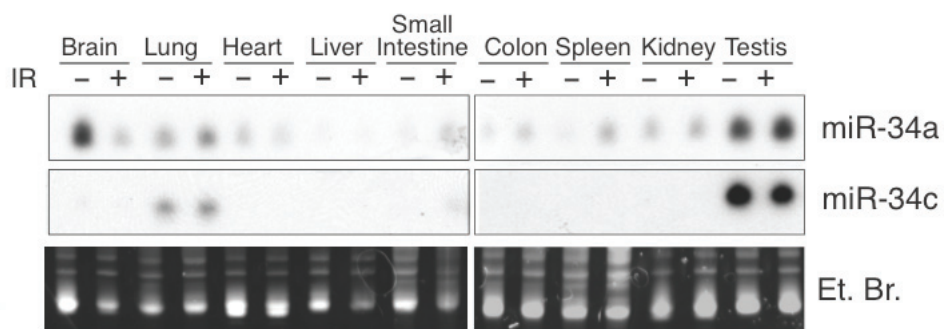

B

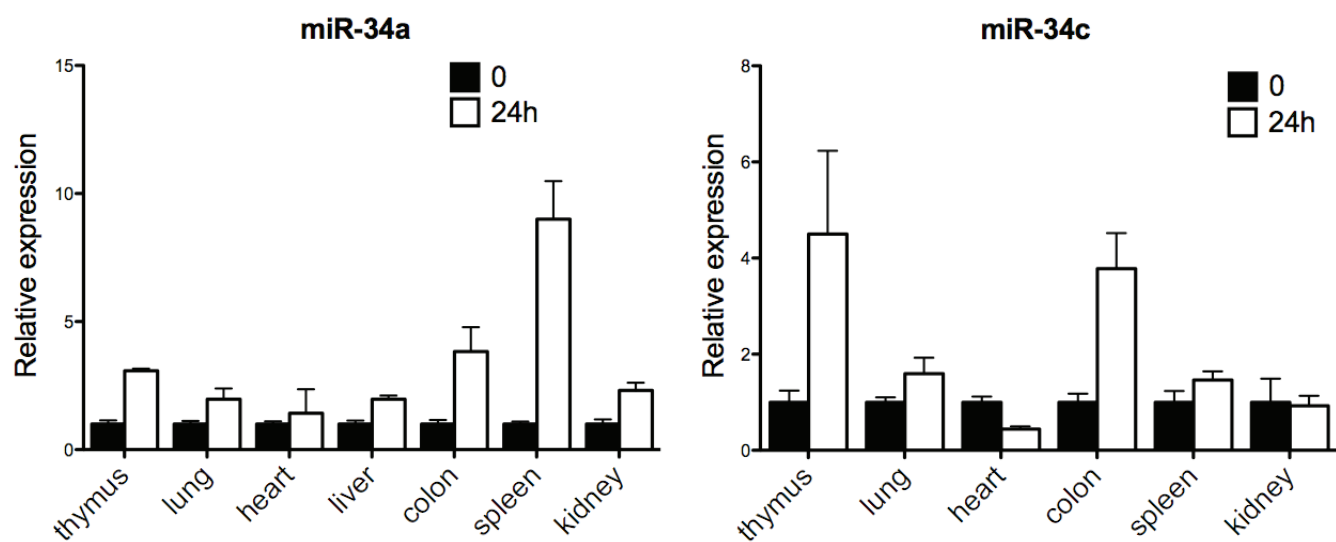

Supplementary Figure 1

Supplement: Figure S1 — Relative miR-34 expression in mouse tissues upon irradiation. (A) MiR-34a and miR-34c expression by Northern blotting under basal conditions and 18 hours after irradiation (10 Gy). (B) MiR-34a (left panel) and miR-34c (right panel) expression by qPCR under basal conditions and 24 h after irradiation (10 Gy). Expression levels of treated samples were normalized to untreated samples. Error bars are standard deviations. (PDF) [file pgen.1002797.s001.pdf]

A

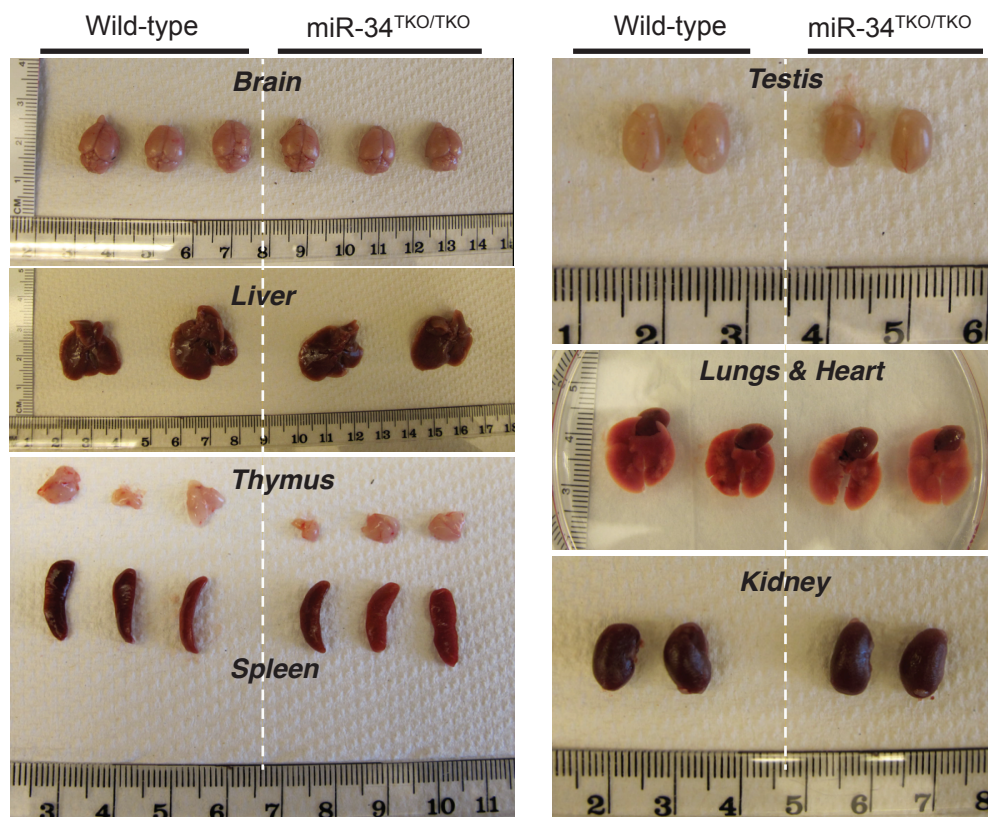

B

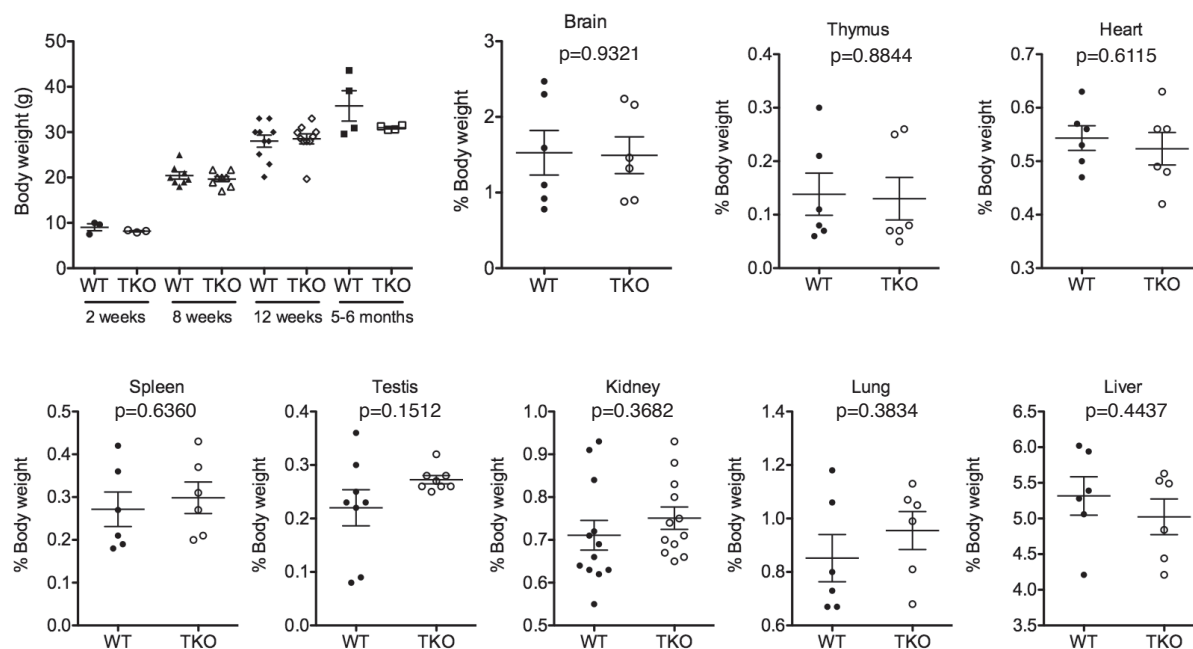

Supplement: Figure S2 — Macroscopic characterization of miR-34TKO/TKO mice. (A) Representative pictures of internal organs obtained from age- and sex-matched wild-type and miR-34-null adult mice. (B) Scatter dot plots showing total body weight of age-matched and sex-matched wild type (WT) and miR-34-null (TKO) mice at different ages (upper-left panel) and relative weight of internal organs (remaining panels; n≥6 per genotype). Error bars indicate mean +/−1 S.E.M. P-values were obtained using the unpaired two-tailed t-test. (PDF) [file pgen.1002797.s002.pdf]

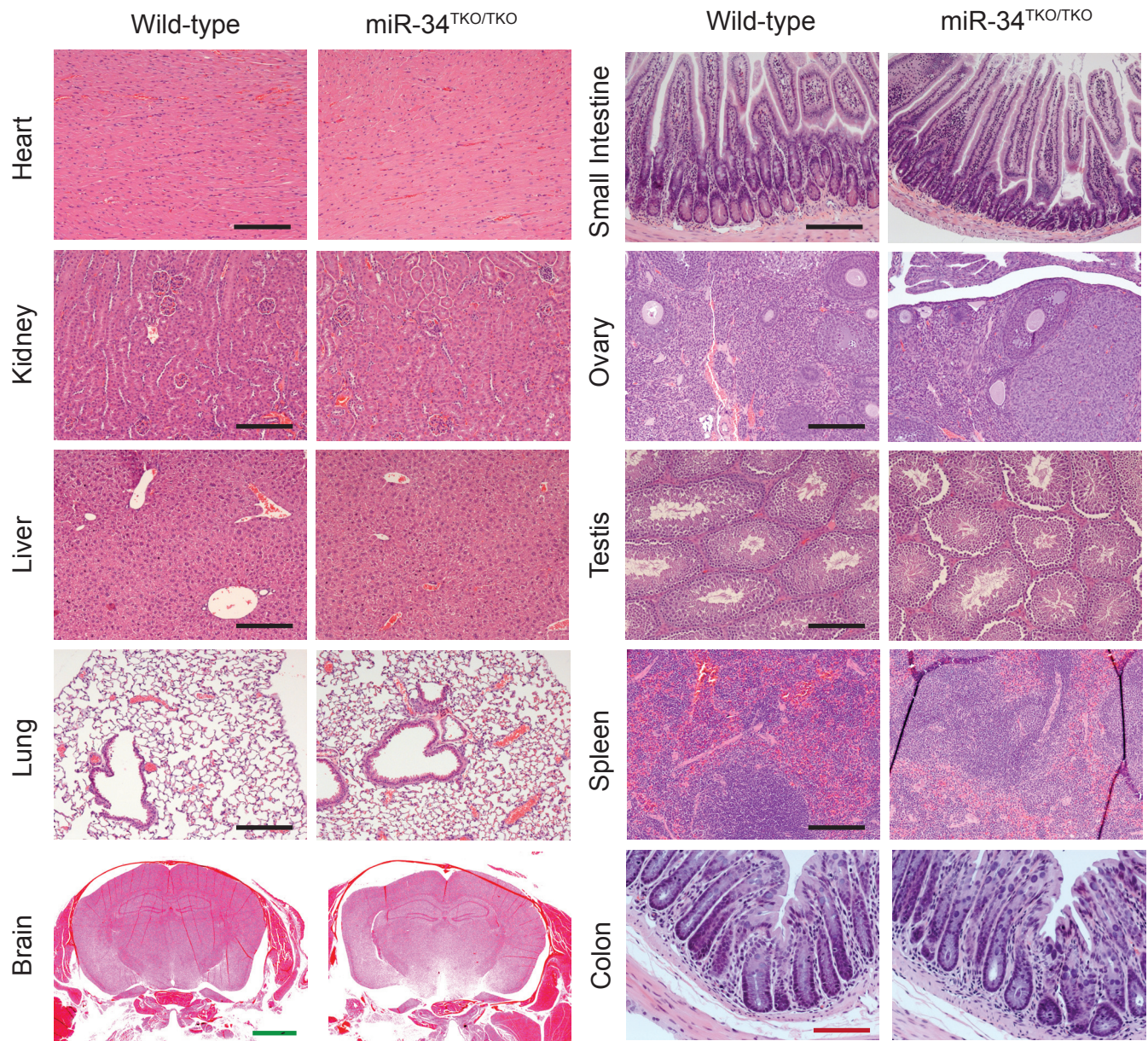

Supplementary Figure 3

Supplement: Figure S3 — Microscopic characterization of miR-34TKO/TKO mice. Representative images of hematoxylin and eosin staining of heart, kidney, liver, lung, small intestine, ovary, testis, and spleen (black scale bar, 200 µm), brain (green scale bar, 2000 µm), and colon (red scale bar, 100 µm) from wild-type and miR-34TKO/TKO mice. (PDF) [file pgen.1002797.s003.pdf]

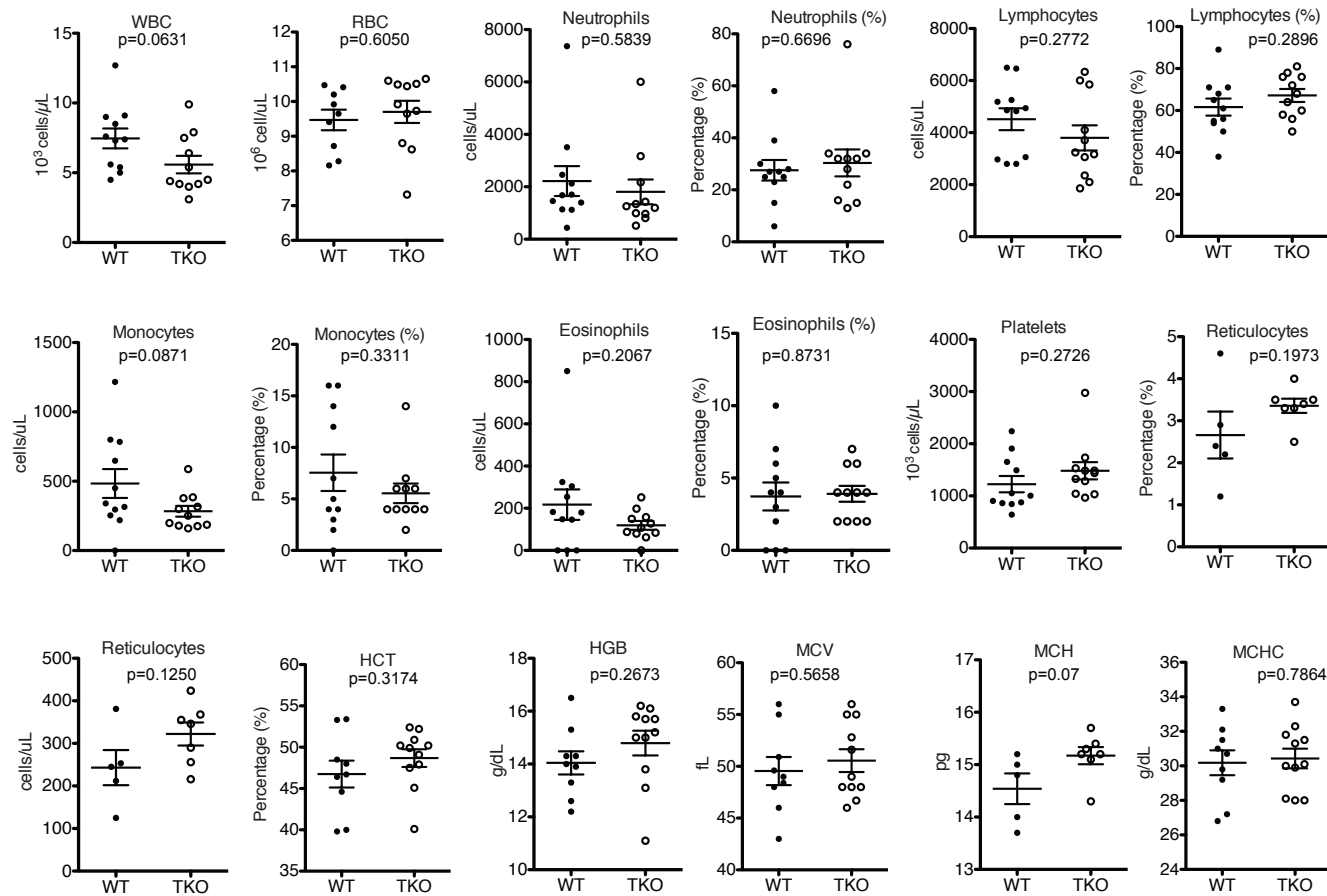

Supplementary Figure 4

Supplement: Figure S4 — Complete blood cell count of age- and sex-matched wild-type and miR-34-deficient mice. Peripheral blood samples obtained from sex- and age-matched adult (age range 3–16 months) wild-type (WT) and miR-34-null (TKO) mice were subjected to complete blood cell count (n≥5 per genotype). Error bars indicate mean +/−1 S.E.M. The P-value for each parameter was calculated using the two-tailed unpaired t-test. Abbreviations used: WBC = White blood cells; RBC = red blood cells; HCT = Hematocrit; HGB = Hemoglobin; MCV = Mean Corpuscolar Volume; MCH = Mean Corpuscolar Hemoglobin; MCHC = Mean Corpuscolar Hemoglobin Concentration. (PDF) [file pgen.1002797.s004.pdf]

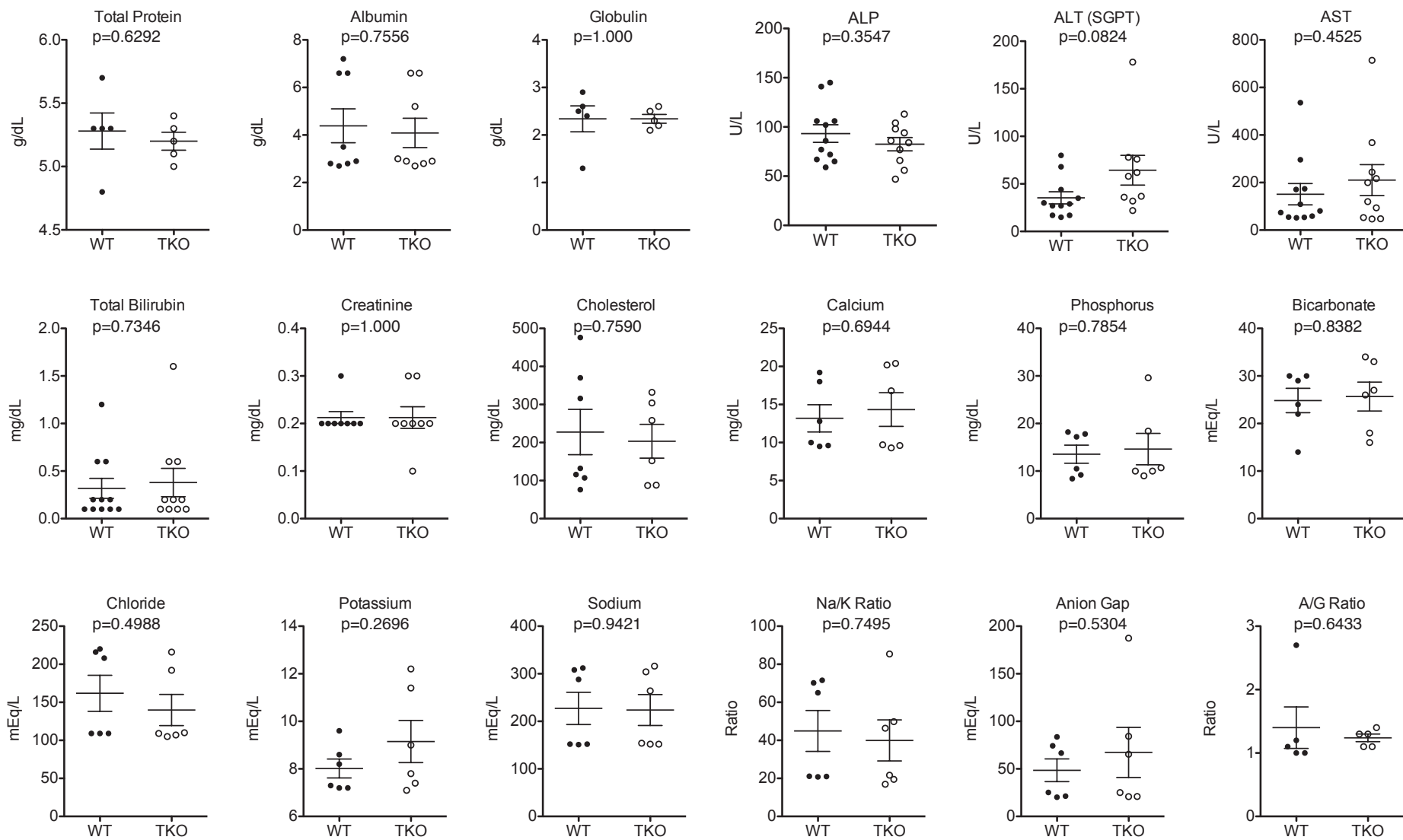

Supplementary Figure 5

Supplement: Figure S5 — Serum chemistry of age- and sex-matched wild-type and miR-34-deficient mice. Samples obtained from sex- and age-matched adult (age range 3–16 months) wild-type and miR-34TKO/TKO mice were subjected to a standard panel of serum chemistry tests to determine liver and kidney function (n≥5 per genotype). Error bars indicate mean +/−1 S.E.M. The P-value for each assay was calculated using the two-tailed unpaired t-test. Abbreviations used: ALP = Alkaline Phosphatase; ALT(SGPT), Alanine Transaminase; AST = Aspartate transaminase; A/G Ratio = (Albumin/Globulin Ratio). (PDF) [file pgen.1002797.s005.pdf]

A

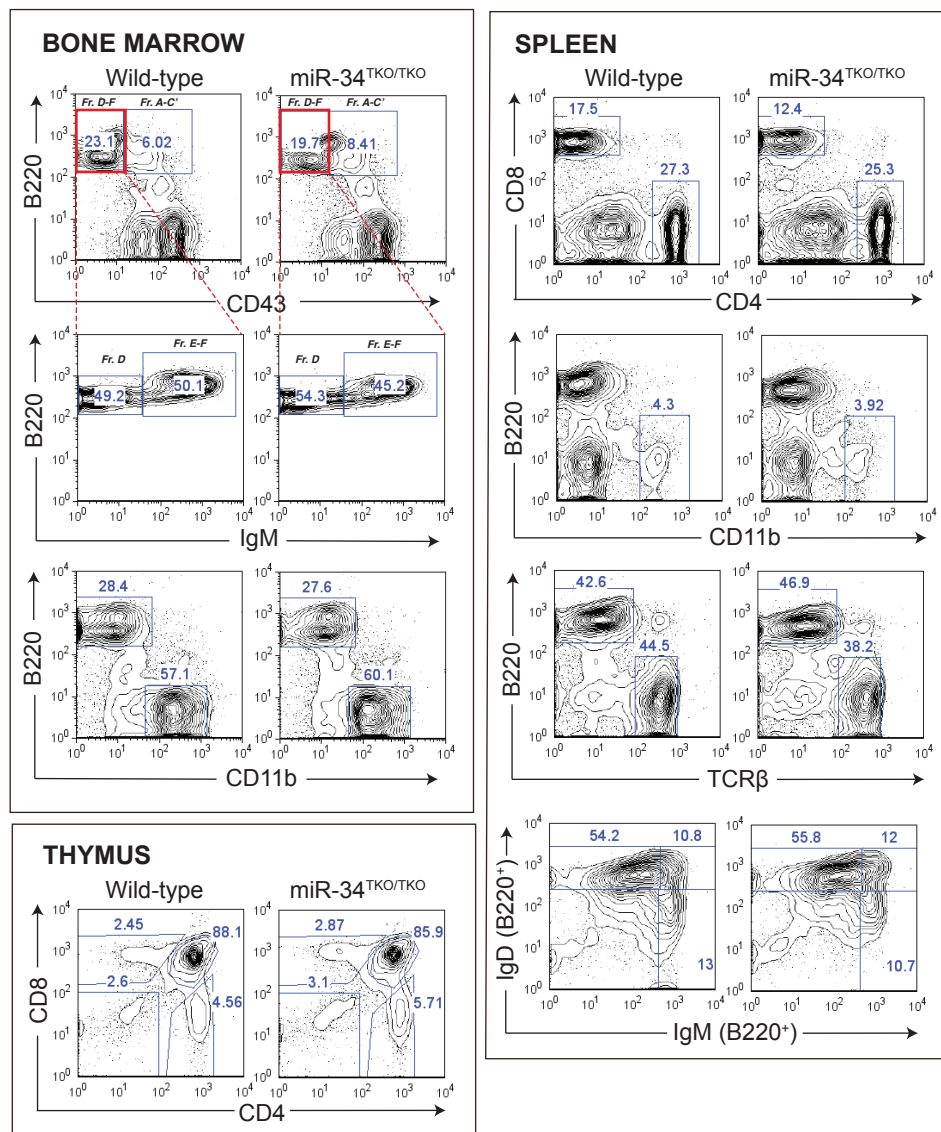

B

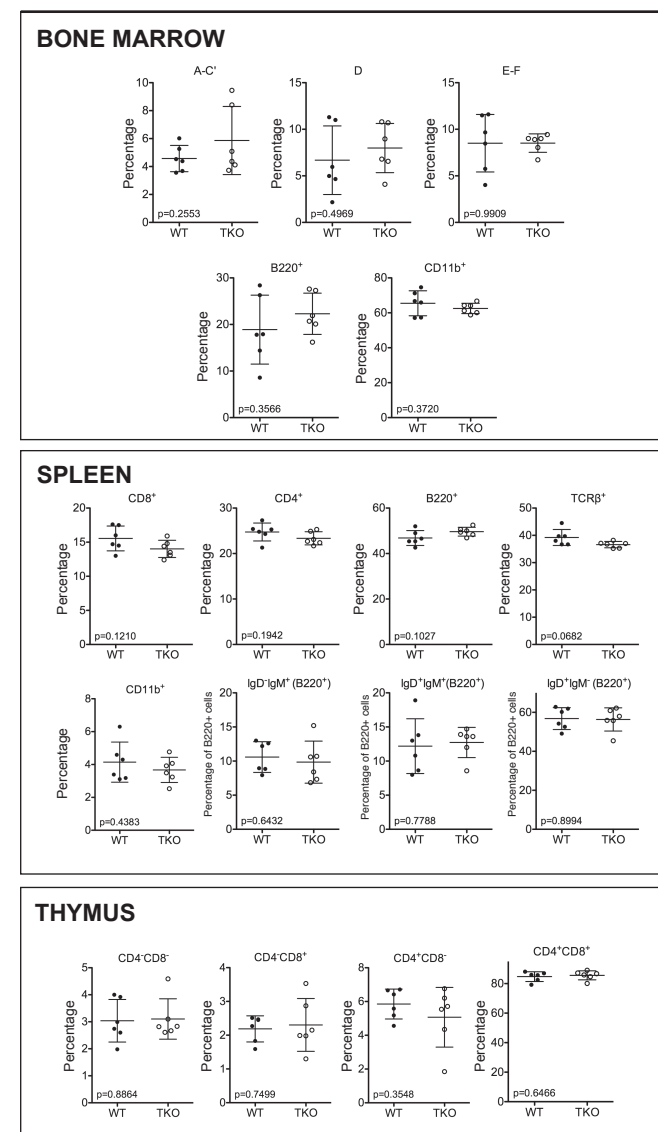

Supplementary Figure 6

Supplement: Figure S6 — Bone marrow, spleen and thymus analysis of age- and sex-matched wild-type and miR-34TKO/TKO mice. (A) Representative contour plots showing lymphoid and myeloid cell populations in the bone marrow, spleen, and thymus of age-matched and sex-matched wild-type (n = 6) and miR-34TKO/TKO mice (n = 6). (B) Scatter dot plots summarizing the results of the analyses shown in (A). Error bars are +/−1 standard deviation. P-values were calculated using the two-tailed t-test. (PDF) [file pgen.1002797.s006.pdf]

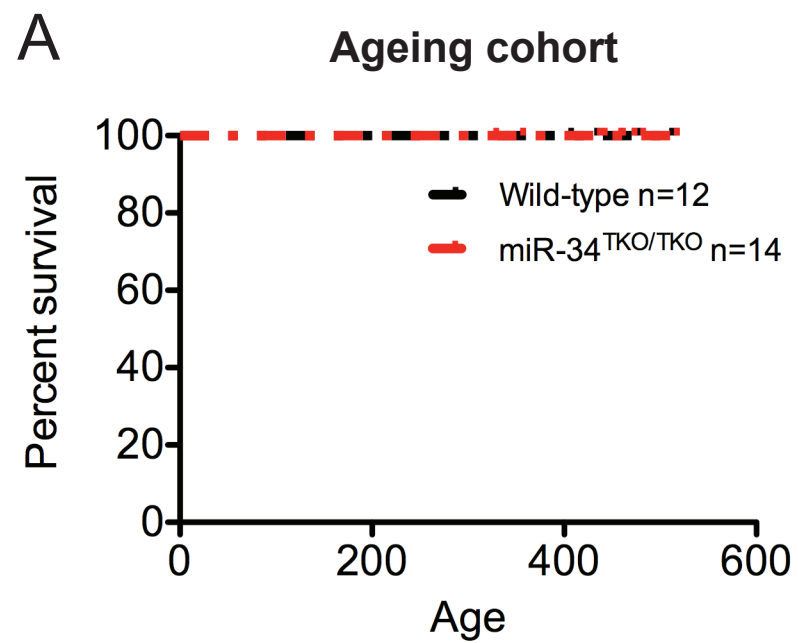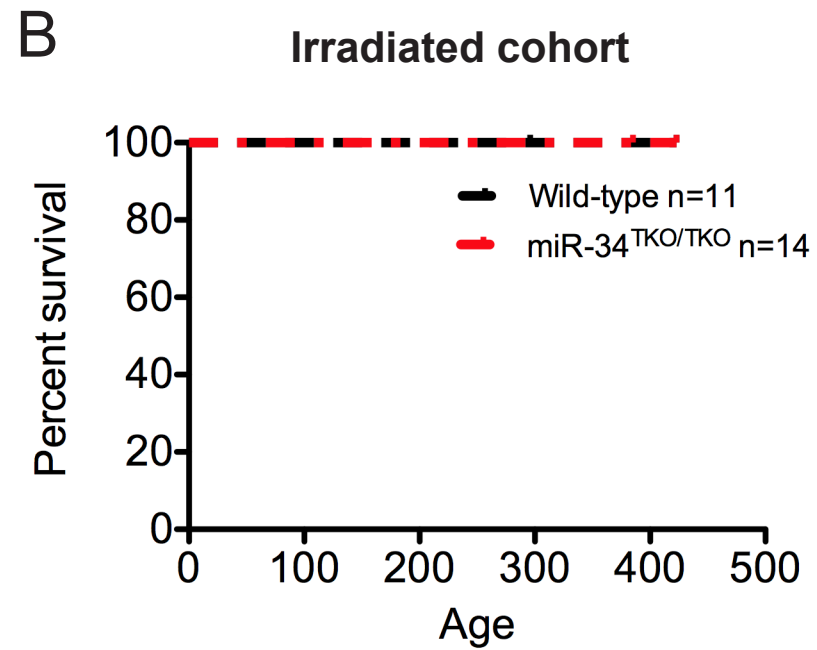

Supplementary Figure 7

Supplement: Figure S7 — Overall survival of wild-type and miR-34TKO/TKO cohorts. (A) Survival curves for wild-type and miR-34TKO/TKO mice. Age range of the cohorts is 359–521 days (mean: 464 days) for wild-type and 359–521 days (mean: 445 days) for miR-34TKO/TKO. P-value = 1 (log-rank test). (B) Survival curves for mouse cohorts with indicated genotypes irradiated with 1 Gy 2 days after birth. Age range of the cohorts is 298–425 days (mean: 333 days) for wild-type and 387–425 days (mean: 401 days) for miR-34TKO/TKO. P-value = 1 (log-rank test). (PDF) [file pgen.1002797.s007.pdf]

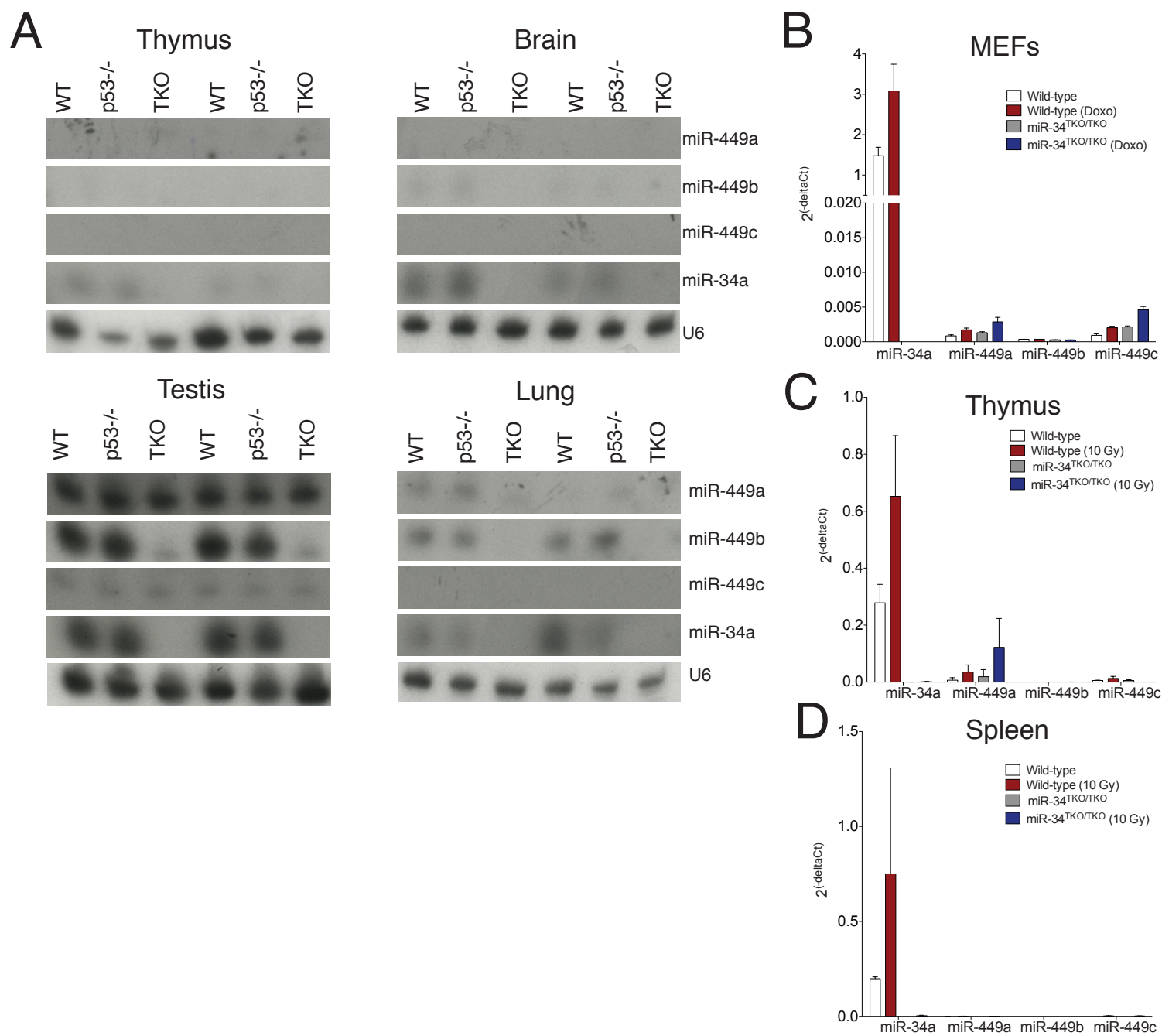

Supplementary Figure 8

Supplement: Figure S8 — Expression of miR-449a, miR-449b and miR-449c. (A) Northern blot detection of miR-449a, miR-449b, miR-449c and miR-34a in a panel of tissues from wild type and miR-34TKO/TKO mice. For each tissue, the same membrane was serially probed first for the three members of the miR-449 family and lastly for miR-34a. RNAs from miR-34TKO/TKO tissues were included to control for cross-hybridization. Notice the loss of signal for miR-449b in the miR-34TKO/TKO lung and testis samples, which likely reflects cross-hybridization of the miR-449b probe to miR-34. (B–D) qPCR detection of miR-449 family members in MEFs (B), thymus (C), and spleen (D) of wild-type, p53−/− and miR-34TKO/TKO mice exposed to DNA damaging agents. MEFs were treated with 0.2 µg/ml doxorubicin for 12 hours. Mice were irradiated with 10Gy and euthanized 6 hours later. Notice that the all three members of the miR-449 family are detected at significantly lower levels compared to miR-34, consistent with the Northern blot analysis shown in panel A. (PDF) [file pgen.1002797.s008.pdf]
