# Supplementary material for: Intact p53-Dependent Responses in miR-34–Deficient Mice
Source: PLoS Genet. 2012 Jul 26;8(7):e1002797. doi: 10.1371/journal.pgen.1002797 (PMC3406012; doi:10.1371/journal.pgen.1002797)
Supplement: Text S1 — Supporting information texts. Genotyping protocols and supplementary methods are provided. (PDF) [file pgen.1002797.s009.pdf]

Text S1

Genotyping

miR-34a

Genotyping primers

AV208	ACAAGACCCTCACCTGAATG
AV209	ATACCCTGGATCTCCAACAG
AV232	GCCATCCTGTTGAGGGACTA

PCR protocol (35 cycles)

Denaturation	95C	45’’
Annealing	57C	1’
Extension	72C	1’

Expected sizes

Allele	Size (bp)	Primer pair
Wild-type	174	AV208 x AV209
Flox	297	AV208 x AV209
Delta	496	AV209 x AV232

miR-34b~c

Genotyping primers

AV338	GCTTGCGGGAAGAAGGACTCG
AV339	ATGACTTTACGGGGTTGACAG
AV370	AAATTCCTCCGACTGAGCCT

PCR protocol (35 cycles)

Denaturation	95C	45''
Annealing	58C	1'
Extension	72C	1'

Expected sizes

**Allele**	**Size (bp)**	**Primer pair**
Wild-type	377	AV338 x AV370
Delta	249	AV338 x AV339

Mouse Tissues

Bone marrow cells were collected by flushing the femur and tibia with PBS+2% FBS. Splenocyte and thymocyte cell suspensions were prepared by meshing the tissues through a cell strainer. Red blood cells were lysed using the ACK lysis buffer (Loundon) before staining for flow cytometry analysis.

Flow Cytometry Analysis and Antibodies

Single-cell suspensions prepared from mouse tissues (bone marrow, thymus and spleen) were incubated in PBS+ 1% FBS + 0.02% NaN_2 containing antibodies raised against the following cell surface markers: B220, IgD, CD8, CD11b, CD25, CD43, CD44 (BD PharMingen), and IgM, TCRb, CD4 (eBioscience). Flow cytometry was performed using a FACSCalibur (BD) and FACSAria (BD). FACS data were analyzed with the FlowJo software (TreeStar).

qPCR and Northern blotting for miR-449 family members

MEFs were left untreated or incubated with 0.2 µg/ml doxorubicin (resuspended in water) for 12 hours. Mice were mock-treated or irradiated with 10 Gy and euthanized 6 hours later. qPCR was performed using Taqman assays purchased from Applied Biosystems according to manufacturer's instructions. Sno-135 amplification was used for normalization. RNA extraction was performed by homogenizing tissues and cells in TRIzol reagent (Invitrogen) according to manufacturer's instructions. For Northern blotting, 10 µg of each RNA sample was loaded into a 15% Urea-PAGE gel and blotted onto a Hybond-N^+ nylon membrane (GE Healthcare). The blots were then serially hybridized with ^{32}P-labeled probes specific for miR-449a, miR-449b, miR-449c, miR-34a and U6. Membranes were stripped by washing twice with pre-warmed 0.1% SDS and allowed to cool at room temperature. The membranes were then exposed to film to ensure complete stripping and finally re-hybridized for other detection. Sequences of probes used are listed below.

miR-449a	ACCAGCTAACAATACACTGCCA
miR-449b	GCCAGCTAACAACACTGCCT
miR-449c	CCAGCTAGCAATGCACTGCCT
miR-34a	ACAACCAGCTAAGACACTGCCA
miR-34c	GCAATCAGCTAACTACACTGCCT

Complete blood count and serum chemistry

CBC, serum chemistry, and phenotypic analyses were performed by the Laboratory of Comparative Pathology in MSKCC.
